# Noncommunicable diseases associated with bleeding disorders, hospitalization, and mortality in patients with dengue in Mexico: A national analysis of confirmed cases in 2024

**DOI:** 10.1371/journal.pntd.0012933

**Published:** 2025-07-28

**Authors:** Diego Rolando Hernández-Galdamez, Miguel Ángel González-Block, Daniela Karola Romo-Dueñas, Erick Antonio Osorio-López, Rosalba Cerón-Meza, Pablo Méndez-Hernández

**Affiliations:** 1 Escuela de Salud Pública de México, Cuernavaca, Morelos, México; 2 Universidad Anáhuac, Ciudad de México, México; 3 Instituto Nacional de Salud Pública, Cuernavaca, Morelos, México; 4 Evisys Consulting, Ciudad de México, México; 5 Centro de Estudios en Salud, Universidad del Valle de Guatemala, Ciudad de Guatemala, Guatemala; 6 Facultad de Medicina, Universidad Nacional Autónoma de México, Ciudad de México, México; 7 Facultad de Agrobiología, Universidad Autónoma de Tlaxcala, Tlaxcala, México; 8 Hospital General de Tlaxcala, Secretaría de Salud de Tlaxcala, Tlaxcala, México; University of Dhaka, BANGLADESH

## Abstract

**Background:**

In Mexico, some of the most prevalent noncommunicable diseases (NCDs) among adults are diabetes, hypertension, and chronic kidney disease (CKD). Mexico is currently facing a syndemic characterized by the convergence of dengue and NCDs. This study aims to describe and analyze the association between the prevalence of NCDs and hospitalization, the presence of hemorrhagic disorders, and death in all officially confirmed cases of dengue in Mexico during 2024.

**Methodology/principal findings:**

This cross-sectional study was carried out through a secondary analysis of the confirmed cases of dengue reported in 2024. We assessed the associations between NCDs and the probability of hospitalization, bleeding disorders, and death, using one logistic regression model for each clinical outcome. We adjusted the three models for age, sex, social security affiliation, ethnicity and for each of the NCDs (diabetes, hypertension, chronic kidney disease, immunosuppression, cirrhosis, and peptic ulcer disease). The most common noncommunicable diseases were diabetes, hypertension, CKD, and immunosuppression. For hospitalization, CKD had the strongest association (OR 5.74), followed by immunosuppression (OR 2.84), peptic ulcer disease (OR 2.33), and diabetes (OR 2.10). We found significant associations between bleeding disorders and several NCDs (diabetes, peptic ulcer disease, immunosuppression, cirrhosis, and hypertension) compared to people without these conditions. People with CKD, peptic ulcer disease and diabetes, had more odds for death compared to those without these comorbidities.

**Conclusions/significance:**

We found a significant association between several comorbidities and worse clinical outcomes in patients with dengue, such as hospitalization, bleeding disorders, or death. The syndemic of NCDs and dengue in Mexico has been rapidly increasing, and this problem needs to be addressed. This work confirms and extends the findings of previous studies and suggests that patients with these comorbidities have worse clinical outcomes.

## Author summary

Dengue infection has shown a significant increase over the last decade, with 2024 marking a record number of cases across Latin America. Although dengue is considered an endemic disease in the region, the magnitude of reported infections in recent years is unprecedented. In Mexico, this trend reflects the regional surge reporting an unprecedentedly elevated number of cases for 2024. Despite typically having low rates of severe illness and case fatality, dengue outcomes can be exacerbated by the presence of underlying noncommunicable diseases. Furthermore, Mexico is facing growing prevalences of noncommunicable diseases such as diabetes, hypertension, and chronic kidney disease. Having a noncommunicable disease as an underlying condition triggers severe illness, including bleeding disorders and higher mortality. This study provides information about the association between the prevalence of noncommunicable diseases and the presence of three different clinical outcomes in dengue-confirmed cases. These findings emphasize the critical necessity of addressing the syndemic interplay between dengue and noncommunicable diseases in Mexico.

## Introduction

Dengue is a viral infection caused by Dengue flavivirus (DENV), which belongs to the RNA virus family *Flaviviridae*. DENV is commonly transmitted to humans by mosquitoes, specifically *Aedes aegypti* [1]. These mosquitoes are spreading their geographic range due to climate change and global warming, posing new challenges for disease control in areas that were previously free from dengue [2]. Dengue is considered a neglected tropical infection with high endemicity, especially in Latin America [3]. According to the Pan American Health Organization (PAHO), 2024 is outlined to record the highest number of dengue cases since 1980. In Latin America, this represents a 70% increase in reported confirmed cases from 2023 (2,053,822) to 2024 (6,916,684). In Mexico, confirmed cases increased by 126.3% in confirmed cases from 54,406 in 2023–123,142 in 2024 [4]. Brazil has the highest incidence in the Americas [5].

Severe dengue (SD) is a complication that generally occurs when the fever subsides and is characterized by symptoms such as intense abdominal pain, persistent vomiting, bleeding from the gums or nose, vomiting blood or blood in the stool, among other symptoms. These are known as warning signs (WS) and require immediate medical attention, which may or may not involve hospitalization [6]. Previous studies have identified several characteristics that may serve as predictors of severe dengue. A 2021 meta-analysis including studies from the Americas identified children, secondary infection, diabetes, and renal disease as associated factors for developing severe dengue [7]. Another study considers chronic kidney disease (CKD) as a high-risk factor for developing WS and SD, particularly in patients with end-stage renal disease on maintenance dialysis [8].

Mexico is currently experiencing a syndemic involving dengue and several NCDs. In the Mexican context, the prevalence of NCDs among adults is diabetes (18.3%), hypertension (29.9%), and CKD (9.11%) [9–11]. There is solid evidence that the presence of pre-existing NCDs contributes to more severe illness and increases the risk of mortality in individuals with severe dengue [12,13]. A recent study in México identified an association between NCDs (diabetes and CKD) and mortality [14].

Identifying factors associated with severe dengue disease is useful in prioritizing interventions to prevent dengue transmission, as well as urgent measures for NCDs prevention and control [13]. This study aims to describe and analyze the association between the prevalence of NCDs and three clinical outcomes -hospitalization, bleeding disorders, and death- among all official confirmed dengue cases reported in Mexico during 2024.

## Methods

### Databases and data extraction

This cross-sectional study is based on a secondary analysis of confirmed dengue cases reported by the Mexico´s Federal Ministry of Health (MoH), using an anonymized, open-access database published by the Directorate General of Epidemiology [15].

**Fig 1:**
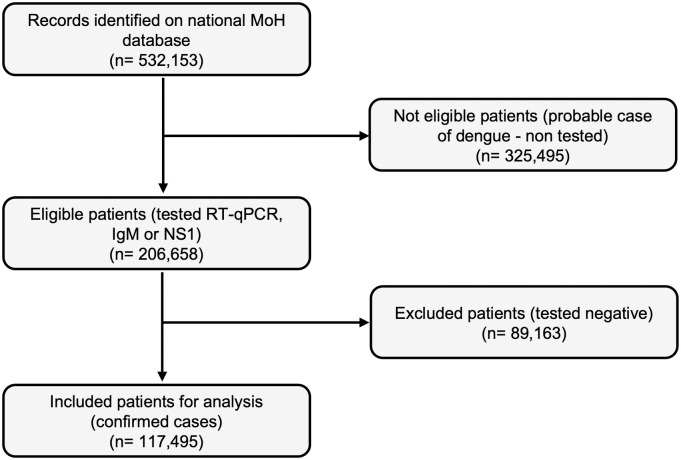
Flowchart of dengue cases selection from National Ministry of Health database, Mexico, 2024. The chart shows the number of ineligible cases, excluded patients, and the total number of confirmed patients included in the analysis. (RT-qPCR = Quantitative reverse transcription polymerase chain reaction; IgM = Immunoglobulin M; NS1 = Non-structural protein 1).

From January 1 to December 4, 2024, the MoH database reported 117,495 laboratory-confirmed dengue cases (Fig 1). The following variables were extracted and analyzed. For sociodemographic status: age, sex, ethnicity, and social security affiliation. For NCDs as independent variables: diabetes, hypertension, chronic kidney disease, immunosuppression, cirrhosis, and peptic ulcer disease. For clinical outcome variables: hospitalization, presence of hemorrhagic disorders, and death. The database does not specify the classification criteria for comorbidities. Data on these conditions were collected through a dichotomous questionnaire completed by the interviewer, based on information provided by the patient directly.

This study was conducted in accordance with the ethical principles outlined in the Declaration of Helsinki. Ethical review was not required, as the analysis was based on publicly available, anonymized data from the Mexican Ministry of Health.

### Statistical analysis

Categorical variables were described as percentages. χ2 and Fisher’s exact tests were performed to compare the proportions of patients with and without noncommunicable diseases against the proportions of patients with and without hospitalization, bleeding disorders, and death. We estimated three separate logistic regression models to evaluate the associations between NCDs and the odds of hospitalization, bleeding disorders and death. Each model corresponded to one clinical outcome and was adjusted for age, sex, social security enrollment, ethnicity, and the presence of specific NCDs (diabetes, hypertension, chronic kidney disease, immunosuppression, cirrhosis, and peptic ulcer disease). Multicollinearity diagnostics were performed using Variance Inflation Factors (VIF), with all values below the conventional threshold, indicating no significant multicollinearity. The Hosmer–Lemeshow test was deemed inappropriate given the large sample size (n = 117,495), as the test is known to over-reject well-fitting models in large datasets. We reported adjusted odds ratios (aORs) with their respective 95% CIs. All statistical analysis was performed using Stata MP software, version 17.0 (Stata Corporation, College Station, TX, USA).

## Results

Of the total confirmed dengue cases in 2024, 55.66% were women. The age group with the highest concentration of cases was adults aged 20–44 (41.83%), followed by adolescents (26.52%). Only 2.42% of reported cases occurred among Indigenous people. A total of 46.04% of the population had social security coverage (IMSS, ISSSTE, Sedena, Pemex, or Semar). The most common NCDs were diabetes (2.97%), hypertension (2.47%), CKD (0.26%), and immunosuppression (0.17%) (Table 1).

**Table 1:** General characteristics of patients with dengue and condition of hospitalization, bleeding disorders, and mortality in Mexico, 2024.

Variables	Total confirmed cases	In-patient cases	Cases with bleeding disorders	Deceased cases
	n = 117,495	n = 45,568	n = 1,028	n = 776
	n (%)	n (%)	n (%)	n (%)
**Sex**				
Women	65,401(55.66)	25,913 (56.87)	630 (61.28)	428 (55.15)
Men	52,094 (44.34)	19,655 (43.13)	398 (38.72)	348(44.85)
**Age group**				
< 5	3,077 (2.62)	1,439 (3.16)	4 (0.39)	31 (3.99)
5-9	9,324 (7.94)	4,158 (9.12)	11 (1.07)	52 (6.70)
10-19	31,161 (26.52)	13,987 (30.69)	32 (3.11)	119 (15.34)
20-44	49,154 (41.83)	16,804 (36.88)	245 (23.83)	242 (31.19)
45-59	16,186 (13.78)	5,276 (11.58)	395 (38.42)	112 (14.43)
≥ 60	8,593 (7.31)	3,904 (8.57)	341 (33.17)	220 (28.35)
**Ethnicity**				
Indigenous	2,843 (2.42)	860 (1.89)	32 (3.11)	21(2.71)
No Indigenous	114,652 (97.58)	44,708 (98.11)	996 (96.89)	755 (97.29)
**Social security affiliation**				
With social security	54,089 (46.04)	20,394 (44.76)	548 (53.31)	283 (36.47)
Without social security	63,406 (53.96)	25,174 (55.24)	480 (46.69)	493 (63.53)
**Comorbidity**				
Diabetes	3,488 (2.97)	1,930 (4.24)	461 (44.84)	123 (15.85)
Hypertension	2,906 (2.47)	1,527 (3.35)	22 (2.14)	81 (10.44)
Chronic kidney disease	310 (0.26)	253 (0.56)	24 (2.33)	33 (4.25)
Immunosuppression	196 (0.17)	131 (0.29)	13 (1.26)	6 (0.77)
Cirrhosis	95 (0.08)	55 (0.12)	6 (0.58)	2 (0.26)
Peptic ulcer disease	59 (0.05)	40 (0.09)	11 (1.07)	5 (0.64)

* χ2 test **Fisher’s exact test.

According to the data analysis, 38.7% (45,568) of the confirmed dengue cases were hospitalized, among which hospitalized women were 56.87%. In the case of hospitalized patients, the most frequent age group was 20–44 years (36.88%), followed by 10–19 years (30.69%). A total of 55.24% of the in-patients’ cases lacked social security affiliation. We found a higher prevalence of NCDs in hospitalized patients compared to outpatients: diabetes (4.24% vs. 2.17%), hypertension (3.35% vs 1.92%), and CKD (0.56% vs 0.08%). It was reported that 0.87% (1,028) of confirmed cases presented bleeding disorders, of which 61.28% were women. In this case, the age groups with the highest incidence of bleeding disorders were adults aged 45–59 (38.42%) and adults over 60 (33.17%). Among those who had hemorrhagic disorders, 44.84% had diabetes, and 2.88% had CKD as the most prevalent comorbidity. No significant differences were found concerning sex or ethnicity. Regarding mortality, 63.53% of them didn´t have a social security affiliation. The age group in which most fatal cases occurred was young adults aged 20–44 (31.19%), followed by older adults (28.35%). Among patients with diabetes and hypertension, we observed higher mortality rates compared to the population without these NCDs (3.53% vs 0.57% and 2.79% vs 0.61%, respectively). The most prevalent comorbidities in patients who died were diabetes (15.85%), hypertension (10.44%), and CKD (4.25%) (Table 1).

Regarding sociodemographic variables, we found significant differences in mortality rates between people with social security and people who did not have social security (36.5 vs 63.5%, respectively). On the other hand, the indigenous population had a lower hospitalization rate in comparison to no indigenous people (30.2% vs 38.9%), despite the similarity in mortality rates between the groups.

**Fig 2:**
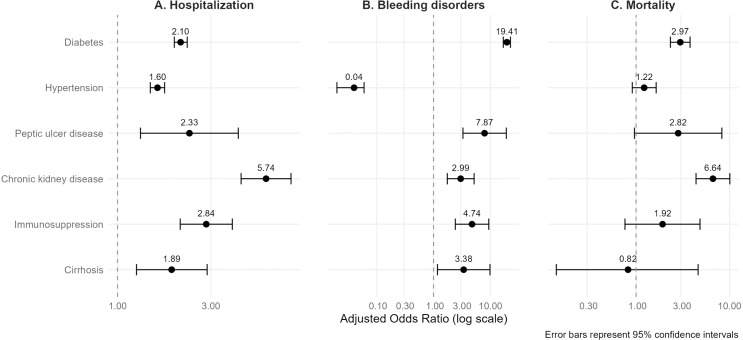
Forest plot of comorbidities association with clinical outcomes on dengue cases in Mexico, 2024. The figure presents adjusted Odds Ratios (aOR) and 95% confidence intervals from three multivariable logistic regression models. Panel (A) shows the association between non communicable diseases (NCDs) and the likelihood of hospitalization. Panel (B) displays the association between NCDs and the likelihood of bleeding disorders, while Panel (C) presents the association between NCDs and mortality. All the models were adjusted for age, sex, social security affiliation, and Indigenous ethnicity. The vertical line represents an aOR of 1.0; an aOR greater than 1.0 indicates a higher odds of the outcome among individuals with the corresponding comorbidity.

Patients with dengue and some comorbidities were more likely associated with worse clinical outcomes, such as being hospitalized, having bleeding disorders, or dying (Fig 2).

For hospitalization, the analysis reported that people with CKD have the highest odds of hospitalization (OR 5.74), followed by immunosuppression (OR 2.84), peptic ulcer disease (OR 2.33), and diabetes (OR 2.10). Only 0.87% of the total dengue cases presented bleeding disorders. Very high odds were found for having bleeding disorders in people with diabetes (OR 19.40), peptic ulcer disease (OR 7.87), immunosuppression (OR 4.74), cirrhosis (OR 3.38), and hypertension (OR 2.99). Regarding mortality, of the total number of confirmed dengue cases, 776 people died (0.66%). For death, only CKD (OR 6.64), peptic ulcer disease (OR 2.82) and diabetes (OR 2.96) are significantly associated whereas hypertension, immunosuppression and cirrhosis are only strongly associated without statistical significance (Table 2). Finally, weidentified that those who presented with bleeding disorders had a higher odds for death (OR 2.39, 95% CI 1.65-3.45, p < 0.001).

**Table 2:** Adjusted odd ratios for NCDs and hospitalization, bleeding disorders, and mortality in dengue-confirmed cases in Mexico, 2024.

Variables	In-patient cases n = 45,568			Cases with bleeding disorders n = 1,028			Deceased cases n = 776		
	Adjusted odds ratio	95% CI	p	Adjusted odds ratio	95% CI	p	Adjusted odds ratio	95% CI	p
**Comorbidity**									
Diabetes	2.10	1.95-2.26	0.000	19.40	16.80-22.42	0.000	2.96	2.32-3.77	0.000
Hypertension	1.60	1.47-1.74	0.000	0.04	0.02-0.06	0.000	1.22	0.91-1.64	0.174
Chronic kidney disease	5.74	4.27-7.70	0.000	2.99	1.73-5.15	0.000	6.64	4.38-10.07	0.000
Immunosuppression	2.84	2.09-3.85	0.000	4.74	2.41-9.33	0.000	1.92	0.76-4.83	0.165
Cirrhosis	1.89	1.24-2.87	0.003	3.38	1.17-9.80	0.025	0.82	0.14-4.61	0.818
Peptic ulcer disease	2.33	1.31-4.14	0.004	7.87	3.27-18.9	0.000	2.82	0.96-8.28	0.059

*Compare without each comorbidity.

Odds ratios were adjusted by age, sex, social security affiliation, and ethnicity for each of the comorbidities analyzed.

## Discussion

In this study, we have found consistent findings concerning previous studies. Our results suggest that the presence of pre-existing NCDs in individuals with dengue is strongly associated with hospitalization, the development of bleeding disorders, or death.

Regarding hospitalization, although not the most prevalent, the NCDs most strongly associated were CKD, immunosuppression, and peptic ulcer disease. For bleeding disorders, the strongest associations were found with diabetes, peptic ulcer disease, and immunosuppression. In the case of mortality, the most strongly associated NCD´s were CKD, diabetes and peptic ulcer disease. This study has several limitations. First, there is a potential for misclassification bias in the recording of NCDs, as the surveillance database includes only dichotomous indicators without specifying diagnostic criteria or validation methods. This limitation may affect the accuracy of the reported associations. Second, although we did not apply formal corrections for multiple testing, each model addressed a distinct clinical outcome based on a priori hypotheses. Still, the potential for type I error should be considered when interpreting the results. Third, regarding the variable “bleeding disorders” as there is no further information on how these causes were classified. The lack of detail prevents us from distinguishing between dengue with warning signs and severe dengue cases, according to Mexican Standards [16]. According to these guidelines, severe dengue is defined by the presence of shock due to plasma leakage, severe bleeding (e.g., hematemesis, melena), or organ failure. Since this level of detail is not available in the database, we interpret the presence of bleeding disorders as a warning sign (WS). We also cannot determine whether these warning signs occurred before or after hospitalization. Nevertheless, this variable may be considered a predictor of severe dengue due to its observed association with death.

Our results are consistent with the findings from other studies that have identified CKD and diabetes as major NCD-related factors for severe dengue and death [7,8,14]. In the study by Rios-Bracamontes et al., for example, our observed ORs are slightly higher for CKD (6.54 vs. 3.35) and diabetes (2.86 vs. 2.07) [14]. Notably, other NCDs such as peptic ulcer disease also play a significant role, being associated with a higher likelihood of hospitalization, bleeding, and death. This may be due to the inherent risk that conditions like peptic ulcer disease and cirrhosis pose for upper gastrointestinal bleeding, which may act synergistically with dengue infection [17]. Therefore, a history of peptic ulcer disease should be considered an important clinical predictor of adverse outcomes.

Although Mexico reports a low case fatality rate (0.66%) compared to other countries in the region, Brazil −despite having the highest incidence of dengue cases in the Americas− has maintained a case fatality rate between 0.04 and 0.09% over the past 10 years [2,18]. This reinforces the need to strengthen for epidemiological surveillance systems, ensuring the early identification and classification of suspected cases based on associated factors to prevent complications. One of the most important early predictors is the presence of WS, with bleeding signs being particularly relevant. According to our study, the NCDs most associated with hemorrhagic disorders are diabetes, peptic ulcer disease, and immunosuppression, in addition to CKD. Additionally, being female and aged over 45 should be considered when identifying high-vulnerable groups, slightly different from the traditionally recognized group of children under 5 years old. Therefore, once a dengue case is confirmed, clinical management should include clinical stratification based on these predictors, and early observation or hospitalization should be considered.

The National Health Program 2020–2024 proposed actions to control dengue infection, considering cultural diversity and a life-course approach. However, it does not identify NCDs as a priority condition to consider during the clinical management of patients with dengue [19]. Nonetheless, PAHO developed the Integrated Management Strategy for the Prevention and Control of Arboviral Diseases, which has been implemented in several Latin American countries. This strategy consists of six components: management, epidemiology, integrated vector management, patient care, laboratory and environment. Regarding patient care, the strategy emphasizes the need to strengthen research on the pathophysiology of dengue under special health conditions [20]. Accordingly, Costa Rica has recognized NCDs as risk factors for severe dengue infection within the PAHO framework [21]. Given the increasing number of cases in recent years, the authors consider that Mexico may also consider the adoption of this strategy, adapting it to national needs, with specific attention to NCD-related vulnerabilities.

## Conclusions

Among the main comorbidities that showed statistical significance in association with the clinical outcomes studied were CKD, peptic ulcer disease, immunosuppression, diabetes, and cirrhosis. The syndemic of NCDs and dengue in Mexico has been rapidly increasing, and this problem needs to be addressed, as other countries in the region have begun to do. This study builds upon previous research findings, demonstrating that patients with these comorbidities tend to have worse clinical outcomes. Further research is needed to better understand the effect of sociodemographic conditions on clinical outcomes in patients with dengue in Mexico.
